# Persistence of African swine fever virus on porous and non-porous fomites at environmental temperatures

**DOI:** 10.1186/s40813-022-00277-8

**Published:** 2022-07-28

**Authors:** Suphachai Nuanualsuwan, Tapanut Songkasupa, Prakit Boonpornprasert, Nutthakarn Suwankitwat, Walaiporn Lohlamoh, Chackrit Nuengjamnong

**Affiliations:** 1 Department of Veterinary Public Health, Faculty of Veterinary Science, Chulalongkorn University, Bangkok, Thailand; 2 Virology Laboratory, National Institute of Animal Health, Chatuchak, Bangkok, Thailand; 3 Department of Animal Husbandry, Faculty of Veterinary Science, Chulalongkorn University, Bangkok, Thailand; 4 Center of Excellence for Food and Water Risk Analysis (FAWRA), Department of Veterinary Public Health, Faculty of Veterinary Science, Chulalongkorn University, Bangkok, 10330 Thailand

**Keywords:** Environmental temperature, Inactivation, African swine fever virus, *D*_T_, Fomite

## Abstract

**Background:**

African swine fever (ASF) is a lethal contagious disease affecting both domestic pigs and wild boars. Even though it is a non-zoonotic disease, ASF causes economic loss in swine industries across continents. ASF control and eradication are almost impossible since effective vaccines and direct antiviral treatment are not available. The persistence of ASFV on fomites plays an important role in the indirect transmission of ASFV to pigs encountering ASFV-contaminated fomites. ASFV persistence on porous and non-porous fomites (glass, metal, rubber, and cellulose paper) at different environmental temperatures was determined. The persistence of ASFV of fomites was determined by the rate of ASFV inactivation in terms of *D*_T,_ or the time required to reduce ASFV per 1 log at each selected environmental temperature (*T*). *D*_T_ is used to compare the persistence of ASFV on the fomites.

**Results:**

The mean *D*_25_, *D*_33_, and *D*_42_, of dried infectious ASFV on glass, metal, rubber, and paper were in the ranges 1.42–2.42, 0.72–1.94, and 0.07–0.23 days, respectively. The multiple *D*_T_ were used to develop a *D*_T_ model to predict the *D*_T_ for some other environmental temperatures. The *D*_T_ models to predict the persistence of dried infectious ASFV on glass, metal, rubber, and paper are log *D*_T_ = (− T/21.51) + 1.34, log *D*_T_ = (− T/20.42) + 1.47, log *D*_T_ = (− T/14.91) + 2.03, and log *D*_T_ = (− T/10.91) + 2.84, respectively. A spreadsheet as a quick and handy tool predicting the persistence time of dried infectious ASFV on fomites at various environmental temperatures based on these *D*_T_ models is available for public to download.

**Conclusion:**

Persistence of dried infectious ASFV on paper are significantly the longest at lower environmental temperatures whereas that of dried infectious ASFV on paper is significantly the shortest at higher environmental temperature.

**Supplementary Information:**

The online version contains supplementary material available at 10.1186/s40813-022-00277-8.

## Background

African swine fever virus (ASFV) is an enveloped double-stranded DNA virus with a genome between 170 and 194 kbp in a virion diameter of 172–191 nm. It belongs to the family *Asfarviridae* and the genus *Asfivirus* [1]. The major clinical symptom of African swine fever (ASF) is a hemorrhagic fever. ASFV morbidity and mortality rates are high and cause a severe threat to the pig industry. A previous study introduced healthy pigs into a pen contaminated with excretions from ASFV-infected pigs. Even though these healthy pigs were infected with ASFV, the infectivity period for indirect transmission was limited [2]. Aside from direct transmission, encountering contaminated fomites plays an important role in indirect transmission of ASFV [3].

Even though ASFV is an enveloped virus, the persistence of ASFV ranges from days to years in animal products and the environment. ASFV is persistent in frozen conditions or at 4 °C for months to years [4]. ASFV persisted in frozen meat and blood for more than 2 years and 6 years, respectively [5, 6]. Some reports indicated that ASFV is persistent for 11–160 days in pig manure [7, 8]. While its stability in pig manure depends upon the storage temperature, a recent study demonstrated that ASFV remains infectious for 8 days at 4 °C and 4 days at 37 °C [9]. Interestingly, the stability of ASFV in manure at environmental temperatures was affected by enzymatic digestion by bacteria [10]. Additionally, ASFV is persistent over a wide pH range between 4 and 11 [11]. Effective thermal inactivation of ASFV occurs at 56 °C for 70 min or at 60 °C for 20 min [12].

ASFV is persistent not only in the environment and pork products but also in feed ingredients. Therefore, either chemical or physical inactivation of imported commodities that are likely to be contaminated with ASFV are among the recommended precautionary risk management measures to control the risk of ASFV introduction to an importing country [13]. ASFV is persistent to a 0.25–2.0% mixture of medium-chain fatty acids consisting of caprylic, capric, and lauric acids while it is only inactivated no more than 1.0 log TCID_50_/ml after being exposed to 2.0% GM in commercial swine feed at room temperature for more than 30 min (*p* < 0.01) [14]. An aqueous formaldehyde-based additive at 0.03% and 0.3% inactivates ASFV titer by 0.8 log TCID_50_/ml and 3.5 log TCID_50_/ml, respectively at room temperature in 30 min inactivation time [15]. ASFV with the initial titer of 7.0 log HAU_50_/cm^3^ was not detectable in complete feed stored between 22–25 and 4–6 °C after 5 and 40 days, respectively [16]. Birch wood served as a model to demonstrate the virucidal activity of citric acid to inactivate dried infectious ASFV on a porous surface [17]. Such scientific reports regarding the persistence or inactivation of ASFV on contaminated fomites are limited. Therefore, the objectives of this study were to determine ASF persistence on glass, metal, rubber, and paper under different environmental temperatures and to develop a *D*_T_ model to predict *D*_T_ of some other environmental temperatures.

## Results

### ASFV persistence on the fomites

**Fig. 1:**
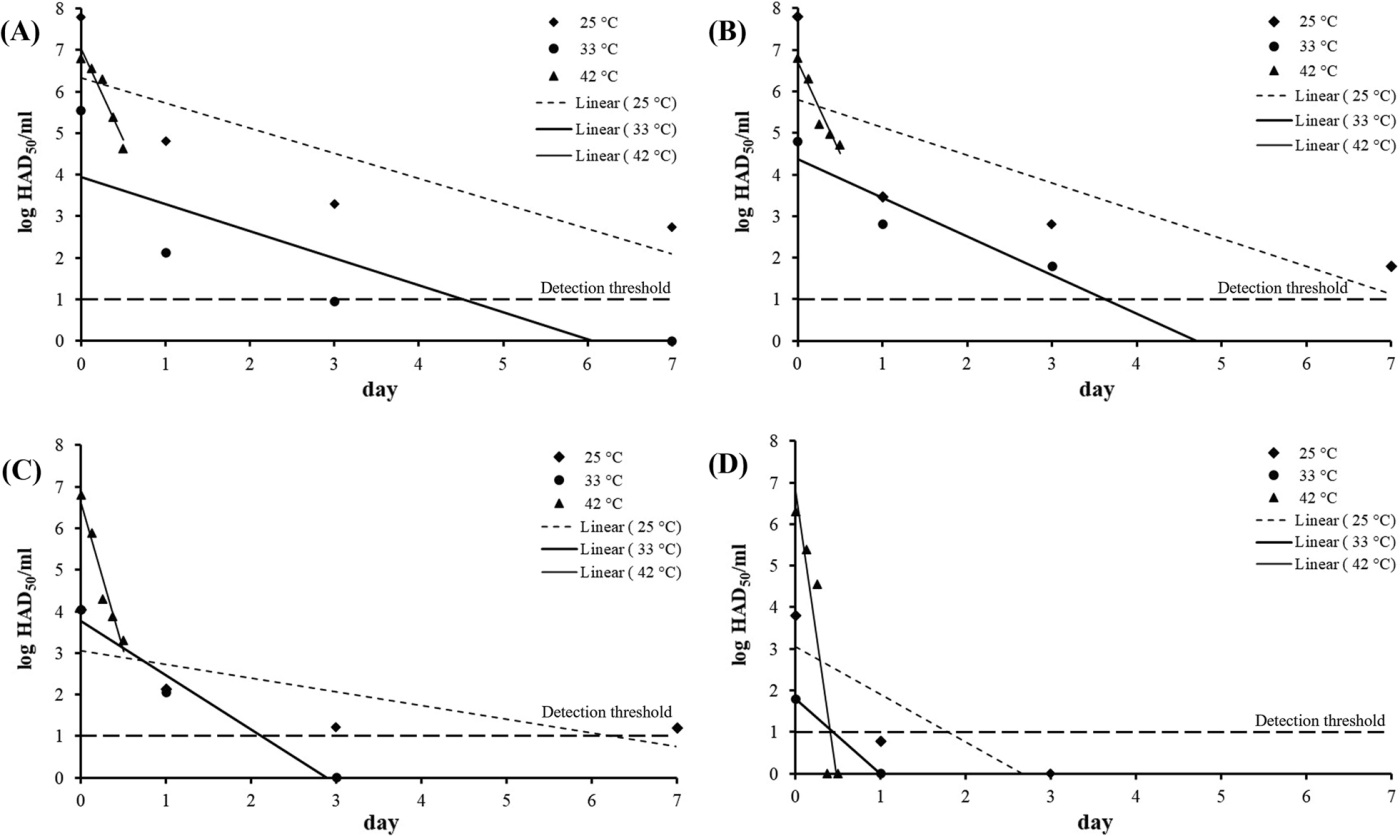
Persistence of dried infectious ASFV on glass **A**, rubber **B**, metal **C** and paper **D** at 25, 33, and 42 °C

The ASFV suspension and blood suspension were spread and dried on the surfaces of the fomites before they were stored at 25, 33, and 42 °C for selected incubation times. The titers of ASFV at time zero were measured by resuspending the dried infectious ASFV on the fomite surface with a cell culture medium. The mean initial titers were in the range 1.8–7.8 log HAD_50_/ml. The titers of dried infectious ASFV on glass, rubber, metal, and paper gradually decreased during storage at environmental temperatures between 25 and 43 °C. Overall, the virucidal effect against dried infectious ASFV was more pronounced on paper than on other fomites at 33 and 42 °C (Fig. 1).

### *D*_T_ of dried infectious ASFV on fomites

The persistence curve was fitted to the linear regression of the log reduction of ASFV titer (*N*_*t*_) versus the incubation time (*t*). The persistence rate of ASFV on the fomite was calculated by fitting the slope of this persistence curve [18]. The best-fit slope of the persistence curve was always negative since the ASFV titers (*y*-axis) supposedly decrease along the incubation time (*x*-axis), indicating the virucidal activity of the drying and temperature effect along time (Fig. 1). The mean *D*_T_, persistence curves, and *gof* of ASFV on four fomites across three environmental temperatures are shown in Table 1. The mean *D*_25_, *D*_33_, and *D*_42_ of dried infectious ASFV of all fomites are in the ranges 1.42–2.42, 0.72–1.94, and 0.07–0.23 days, respectively. The ASFV persistence curves across three environmental temperatures on four fomites are statistically significant (*p* < 0.05). Therefore, in this study, ASFV was inactivated by drying on glass, rubber, metal, and paper and further incubation at environmental temperatures between 25 and 42 °C.

**Table 1:** *D*_T_ and persistence curves of infectious ASFV on several fomites at environmental temperatures

Fomite	Temp. (°C)	*D*_T_ (day)^a^	Inactivation curve^b^	*gof* ^c^		*p* value
				*r*^2^	RMSE	
Non-porous						
Glass	25	1.42 ± 0.05	*log N*_*t*_ = − 0.69*t* + 6.42	0.74	1.20	< 0.001
	33	0.72 ± 0.10	*log N*_*t*_ = − 0.65*t* + 3.95	0.65	1.41	< 0.001
	42	0.23 ± 0.05	*log N*_*t*_ = − 4.40*t* + 7.03	0.85	0.36	< 0.001
Metal	25	1.90 ± 0.10	*log N*_*t*_ = − 0.51*t* + 2.93	0.66	1.08	< 0.001
	33	1.32 ± 0.53	*log N*_*t*_ = − 0.33*t* + 3.05	0.43	1.11	0.02
	42	0.14 ± 0.02	*log N*_*t*_ = − 7.20*t* + 6.63	0.91	0.43	< 0.001
Porous						
Rubber	25	1.54 ± 0.04	*log N*_*t*_ = − 0.67*t* + 5.80	0.61	1.58	< 0.001
	33	1.08 ± 0.01	*log N*_*t*_ = − 0.35*t* + 3.76	0.58	0.87	< 0.001
	42	0.23 ± 0.04	*log N*_*t*_ = − 4.40*t* + 6.70	0.79	0.43	< 0.001
Paper	25	2.42 ± 0.28	*log N*_*t*_ = − 0.42*t* + 2.28	0.48	1.26	0.01
	33	1.94 ± 0.01	*log N*_*t*_ = − 0.17*t* + 0.92	0.32	0.69	0.04
	42	0.07 ± 0.01	*log N*_*t*_ = − 14.39*t* + 6.84	0.87	1.04	< 0.001

^a^Mean ± S.D

^b^ASFV titer (log *N*_t_) at incubation time *t* (day)

^c^goodness-of-fit

Tukey’s multiple comparisons of *D*_T_ of dried infectious ASFV on fomites at 3 environmental temperatures were determined and are shown in Table 2. In general, the environmental temperatures are inversely related to the *D*_T_ of dried infectious ASFV; as the environmental temperature rises, the mean *D*_T_ drops. The mean *D*_25_ of dried infectious ASFV in four fomites is the longest and this is followed by *D*_33_ and *D*_42_, respectively (*p* < 0.05), indicating that warmer environmental temperatures had a shorter *D*_T_ and vice versa. The significant differences in *D*_T_ of dried infectious ASFV on glass and rubber across environmental temperatures indicated that the dried infectious ASFV was inactivated faster at warmer environmental temperatures. The mean *D*_25_ and *D*_33_ of dried infectious ASFV on paper are significantly the longest whereas the mean *D*_42_ of dried infectious ASFV on paper is significantly the shortest. The mean *D*_25_, *D*_33_, and *D*_42_ of dried infectious ASFV on metal are between those of dried infectious ASFV on rubber and paper (*p* < 0.05).

**Table 2:** Comparison of mean *D*_T_ ± S.D. (day) of dried infectious ASFV on 4 fomites at 3 environmental temperatures

Fomite	Environmental temperature (°C)		
	25	33	42
Glass	1.42 ± 0.05^A,a^	0.72 ± 0.10^A,b^	0.23 ± 0.05^A,b,c^
Rubber	1.54 ± 0.04^B,a^	1.08 ± 0.01^B,b^	0.23 ± 0.04^A,c^
Metal	1.90 ± 0.10^C,a^	1.32 ± 0.53^B,C,a^	0.14 ± 0.02^B,b^
Paper	2.42 ± 0.28^D,a^	1.94 ± 0.01^C,a^	0.07 ± 0.01^C,b^

In the column-wise comparison, mean *D*_T_ with different letters implies that there are statistically significant differences (*p* < 0.05) among the different fomites for the same environmental temperature. (letters A through D). In the row-wise comparison, mean *D*_T_ with different letters implies that there are statistically significant differences (*p* < 0.05) among the different environmental temperatures for the same fomite (letters a through c)

### *D*_T_ model

**Fig. 2:**
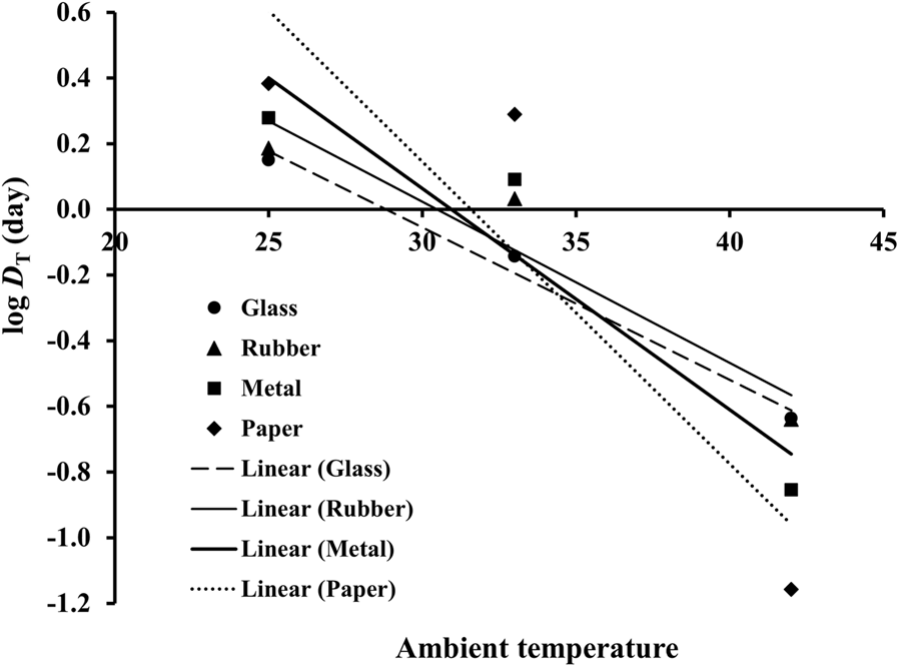
DRT curves were fitted to the log *D*_T_ of dried infectious ASFV on fomites

Based on the *D*_T_ of dried infectious ASFV in Table 1, the DRT curves were drawn from the logarithmic *D*_T_ of dried infectious ASFV on fomites on the *y*-axis versus the environmental temperatures on the *x*-axis (Fig. 2). The mean and 95% CI of *z* values and the predicted *D*_T_ models on fomites are shown in Table 3. The *gof* values of the *D*_T_ models of all fomites indicate that the *D*_T_ models could be used to predict the *D*_T_ for some other environmental temperatures.

**Table 3:** *Z* value and *D*_T_ model of dried infectious ASFV for 4 fomites

Fomite	*z* value (°C)		*D*_T_ model^a^	*gof*		*p* value
	Mean	95% CI		*r*^2^	RMSE	
Glass	21.51	18.28–26.13	log *D*_T_ = – $\left( {\frac{T}{21.51}} \right)$ + 1.34	0.96	0.07	< 0.001
Rubber	20.42	15.71–29.16	log *D*_T_ = – $\left( {\frac{T}{20.42}} \right)$ + 1.47	0.92	0.12	< 0.001
Metal	14.91	11.42–21.45	log *D*_T_ = – $\left( {\frac{T}{14.91}} \right)$ + 2.03	0.91	0.14	< 0.001
Paper	10.91	7.76–18.36	log *D*_T_ = – $\left( {\frac{T}{10.91}} \right)$ + 2.84	0.86	0.32	< 0.001

^a^log *D*_T_ (day) for the unknown environmental temperature *T* (ׄ°C)

## Discussion

In this study, the persistence of the African swine fever virus (ASFV) on porous and non-porous fomites at environmental temperatures (25, 33, and 42 °C) was investigated. The porous fomites were rubber and paper while the non-porous fomites were glass and metal. The incubation times were designated to be able to follow the reduction of dried infectious ASFV titers. The persistence of dried infectious ASFV was longer at a lower environmental temperature for both porous and non-porous fomites (Fig. 1). At 42 °C, the persistence of dried infectious ASFV on all fomites lasted only one day. At 25 °C, the persistence of dried infectious ASFV on glass, rubber, metal was longer than 7 days from the initial titer in the range 4–7 log HAD_50_/ml, while that of dried infectious ASFV on paper was only 3 days from the initial titer (only 3.8 log HAD_50_/ml) at the same temperature. The persistence of dried infectious ASFV on fomites at 33 °C with the mean initial tiers in the range of 1.8–5.8 log HAD_50_/ml was between those at 25 and 42 °C (Fig. 1). Interestingly, since the initial titers of dried infectious ASFV on different fomites were not consistent as a result of the virus degradation during storage, the direct comparison of log reduction of ASFV on different fomites could be biased. In this study, the persistence rate in terms of *D*_T_ [19] was used to comparatively determine the persistence of dried infectious ASFV on different fomites at different environmental temperatures.

The result of this study demonstrates that the mean *D*_25_ and *D*_33_ of dried infectious ASFV on paper were statistically longest while those of dried infectious ASFV on glass were statistically shortest (Table 2). This indicates that at 25 and 33 °C dried infectious ASFV is the most and the least persistent on paper and glass, respectively (Table 2). On the other hand, at 42 °C dried infectious ASFV on paper and glass become the least and the most persistent, respectively. According to the *D*_T_ model in Table 3, a lower *z* value results in a larger change of *D*_T_ and vice versa. The *z* values of dried infectious ASFV on paper and glass are the lowest (10.91 °C) and the highest (21.51 °C), respectively (Table 3). As the environmental temperature rises from 33 to 42 °C, *D*_T_ of dried infectious ASFV on paper drops faster (Table 2). Until *D*_42_ of dried infectious ASFV on paper and glass becomes shortest and longest, respectively, which is opposite to previous values at *D*_25_ and *D*_33_. Note that, considering the overall persistence on the fomites, dried infectious ASFV has a longer persistence on the porous fomites at the lower environmental temperatures assayed (25 and 33 °C) while dried infectious ASFV becomes more persistent on the non-porous fomites at the higher environmental temperature selected (42 °C).

After being infected with ASFV, pigs develop clinical symptoms and ASFV is secreted. Then, ASFV readily contaminates various types of surfaces in the farm environment. This could lead to indirect transmission when pigs encounter such contaminated fomites [20, 21]. Even though ASFV is highly stable in the environment, the likelihood of ASFV transmission depends upon the initial titer of the virus [12, 22]. The highest titer of ASFV, particularly in the blood of infected pigs, is 9 log HAD_50_/ml [20]. This value, including the mean *D*_T_ of dried infectious ASFV on fomites under the environmental temperatures from this study (Table 2) were used to calculate the minimum to maximum persistence of dried infectious ASFV on porous and non-porous fomites as shown in Table 4. This range of the persistence of dried infectious ASFV is useful to determine the safe downtime not only in the farm environment but also in some other environments along the pork supply chain, particularly where cleaning and disinfection are almost impossible.

**Table 4:** Persistence of dried infectious ASFV on non-porous and porous fomites at selected environmental temperatures

Fomite	25 °C		33 °C		42 °C	
	Min (days)	Max (days)	Min (days)	Max (days)	Min (days)	Max (days)
Non-porous	11	17	6	12	1	2
Porous	14	22	10	17	1	2

The aim of this study was to suggest the possible range of persistence of dried infectious ASFV contaminating various fomites in the farm environment against selected environmental temperatures, mimicking seasonal temperatures. For the temperate climate of Thailand, the environmental temperatures were ranged between 25 and 33 °C [23] while the extreme maximum environmental temperature during summer was approaching 42 °C [24]. At 42 °C, the persistence of dried infectious ASFV on both non-porous and porous fomites was comparable and over the range of 1–2 days, while at 25 and 33 °C the persistences of dried infectious ASFV on porous fomites are slightly longer than that on non-porous fomites by 3–5 days. The maximum persistence of dried infectious ASFV was about 2–3 weeks at 25 °C on porous fomites. This indicates that the environmental temperature affects the persistence of dried infectious ASFV more than the kind of fomite.

Even though this study demonstrates the limited persistence period of dried infectious ASFV on various fomites (Table 4), cleaning and disinfection are still mandatory to mitigate the risk of indirect transmission of ASFV through contaminated fomites. Some previous studies reported the ranges of virucidal activity of chemical disinfectants against dried infectious ASFV on porous and non-porous fomites [17, 25]. Both 500 ppm hypochlorite and 1.0% citric acid effectively inactivated dried infectious ASFV on a non-porous fomite with a contact time of 10 min. The log reductions of dried infectious ASFV by 500 ppm hypochlorite with a contact time of 10 min on steel and plastic were 4.80 ± 0.46 and 4.75 ± 0.61 log CCID_50_/ml, respectively, while those of dried infectious ASFV using 1.0% citric acid with a contact time of 10 min on steel and plastic were 4.80 ± 0.11 and 4.88 ± 0.38 log CCID_50_/ml, respectively [25]. This implies that the virucidal activity of chemical disinfectants against dried infectious ASFV appears to be the same among non-porous fomites. On the other hand, for the porous fomite, the log reductions of dried infectious ASFV using 1,000 ppm hypochlorite and 2.0% citric acid with a contact time of 30 min on birch wood were 3.75 ± 0.44 and 4.72 ± 0.41 log CCID_50_/ml, respectively [17]. These two previous studies were performed by the same group of authors with similar experimental designs. Therefore, the results of these two studies were assumed to be comparable. Note that the initial titer of dried infectious ASFV in these two studies was in the range of 5–7 log CCID_50_/ml, therefore applying disinfectants with those concentrations and contact times results in the residual infectivity of ASFV on the fomite. To achieve roughly similar log reductions of dried infectious ASFV, the porous fomite (birchwood) required to double the disinfectant’s concentration comparing with the non-porous fomite assays. The virucidal activities of disinfectant against dried infectious ASFV on non-porous fomite seem to be higher than those on porous fomite. Since the type of fomite could influence not only the virucidal activities of chemical disinfectant but also the persistence of dried infectious ASFV, the choices of the risk mitigation measure should consider the type of fomite.

Since the *D*_T_ models against dried infectious ASFV on porous and non-porous fomites as shown in Table 3 are complicated and prone to error, an easy spreadsheet predicting the *D*_T_ and the persistence time from these *D*_T_ models 4 is provided (Additional file 1). This spreadsheet is intended to expand and ease the field applications by just entering the environmental temperatures including the desire log reduction of ASFV under the fomite worksheet; this spreadsheet promptly provide the lower and upper 95% confidence interval of the persistence time. The link to download this spreadsheet is available.

As far as we are aware, this is the first study of the persistence of dried infectious ASFV on porous and non-porous fomites at environmental temperatures. To know and finely assess the persistence of dried infectious ASFV under environmental temperatures is potentially beneficial to the swine industry to determine the safe downtime from the farm environment to the processing plants in the pork supply chain, particularly where cleaning and disinfection are too difficult.

## Conclusion

The persistence of dried infectious ASFV on porous and non-porous fomites under environmental temperatures was evaluated. The persistence of ASFV was in turn determined by the rate of ASFV inactivation in terms of *D*_T_ or the time required to reduce ASFV infectious titer per 1 log at an environmental temperature (*T*). The mean *D*_25_, *D*_33_, and *D*_42_, of dried infectious ASFV on glass, metal, rubber, and paper were in the ranges 1.42–2.42, 0.72–1.94, and 0.07–0.23 days, respectively. The persistence of dried infectious ASFV was affected by both environmental temperatures and the type of fomite. The *D*_T_ models to predict the persistence of dried infectious ASFV on glass, metal, rubber, and paper are log *D*_T_ = (− T/21.51) + 1.34, log *D*_T_ = (− T/20.42) + 1.47, log *D*_T_ = (− T/14.91) + 2.03, and log *D*_T_ = (− T/10.91) + 2.84. The *D*_T_ values of dried infectious ASFV on glass, metal, rubber, and paper provide insight into the risk of ASFV transmission through contaminated fomites e.g. vehicles, rubber boots, or paper packaging.

## Materials and methods

### Cell preparation

Primary swine macrophages were aseptically collected from 24 week-old crossbred pigs in which the absence of PCV2, CSFV, PRRSV, and ASFV was confirmed by polymerase chain reaction assay (PCR). Peripheral blood morphonuclear cells (PBMCs) were prepared from defibrinated swine blood as previously described [12]. The cells were cultured in autogenous pig serum for maturation and then, after 3–4 days, monocyte-derived macrophages (MDMs), that is macrophage-like round cells, were proliferated on a vessel surface. The cells were continually cultured in RPMI-1640 (Gibco, Waltham, MA, USA) culture medium containing 10% fetal bovine serum (Sigma-Aldrich, St. Louis, MO, USA) and supplemented with antibiotic–antimycotic solution (Gibco, Waltham, MA, USA).

### ASFV titration

The ASFV isolates (Asian epidemic strain, genotype II) were originated from pork products confiscated from international tourists between 2018 and 2020. The ASFV stocks (ASFV-NIAH-BL01-05) for the inactivation studies were routinely maintained and titrated in PBMCs culture and stored in aliquots at − 80 °C until use. All experiments with ASFV were performed at biosafety level 3 at the NIAH.

The viral titers were determined by PBMC cell cultures. Approximately 1.5 × 10^6^ cells/well in 96-well plates were seeded in each well for 3–4 days before the assay. Fifty microliters of a tenfold serial solution of samples were inoculated into the wells in quadruplicate and the samples were incubated in a CO_2_ incubator at 37 °C for 5–7 days. The presence of haemadsorption (HAD) was examined under the microscope and the 50% HAD infectious dose per ml (HAD_50_/ml) was calculated using the Reed and Muench method.

### ASF inactivation on fomites

To study the effect of various environmental matrices, two types of fomites, porous and non-porous, were studied. The porous fomites were silicone rubber and cellulose paper (Whatman® Cat. No. 1030 023) while the non-porous fomites were borosilicate petri dish glass and metal (AISI 304 2B stainless steel). ASFV suspension (500 ul) was dropped onto the rubber, cellulose, and paper and evenly spread on the surface while ASFV-spiked blood was dropped onto the glass. The initial infectious ASFV suspension and blood suspension had titers of 5.0 log HAD_50_/ml and 4.5 log HAD_50_/ml, respectively. All fomite surfaces were air-dried inside a biological safety cabinet at room temperature for 30 min. The air-dried infectious ASFV suspension was incubated at environmental temperatures of 25, 33, and 42 °C in an advanced microbiological incubator (Heratherm IMH 60; Thermo Fisher Scientific, Melbourne, Australia). After reaching the environmental inactivation time, 500 µl of cell culture medium (RPMI–1640) was added to and mixed with the fomite surface The mixture on the fomite surface was carefully scraped and collected. Then the mixture was centrifuged, harvested, and stored at − 80 °C until the residual infectious virus was titrated.

### Persistence curve

The viral persistence rate follows first-order kinetics where a linear persistence curve is fitted to the reduction of log ASFV titer as a function of incubation time at a constant environmental temperature [26–29]. The negative reciprocal of the slope of this linear curve is *D*_T,_ as shown in the following equation:

1 $$\log N_{t} = - \frac{t}{{D_{t} }} + logN_{0}$$

where *N*_t_ and *N*_0_ are the ASFV titer at incubation times *t* and zero, respectively.

### *D*_T_ model

The DRT curve is derived from fitting multiple values of *D*_T_ on a semi-logarithmic scale across environmental temperatures tested. The linear equation of the DRT curve is fitted to log *D*_T_ (DRT) as a function of environmental temperature. This linear equation becomes the *D*_T_ model. Analogous to *D*_T_, the *z* value is the negative reciprocal of the slope of the DRT curve. Therefore, the *z* value is the temperature required to change *D*_T_ by 90%. *D*_T_ for an environmental temperature could be predicted by the *z* value together with the *y*-intercept of the fitted linear equation as shown in the following equation:

2 $$\log D_{T} = - \frac{T}{Z} + {\text{y}} - {\text{intercept}}$$

where

*D*_T_ is the *D* of ASFV at environmental temperatures *T.*

*z* is the negative reciprocal of the slope.

### Statistical analyses

The statistical significance of the persistence curve to determine the ASFV reduction on the fomite surface was obtained using an *F*-test of the regression analysis. Likewise, the statistical significance of the DRT curve to determine the change of persistence rate as a result of the environmental temperature was obtained using an *F*-test of the regression analysis. The goodness-of-fit (*gof*) of both the persistence curve and the DRT curve was determined using the correlation coefficient (*r*^2^) and the root mean square error (RMSE) [27]. The statistical difference of ASFV persistence rates (*D*_T_) across three environmental temperatures was determined using one-way analysis of variance (ANOVA). Likewise, the fomite effect was determined by the statistical difference in persistence rates (*D*_T_) over four types of fomite at the same environmental temperature. After ANOVA indicated a statistically significant difference, Tukey’s multiple comparison test was used to determine the pair-wise *D*_T_ differences in terms of either temperatures or fomites. IBM® SPSS® Statistics version 22 software (SPSS Inc., Chicago, IL, USA) was used for the statistical analyses.

## Supplementary Information

**Additional file 1**. Predicting *D*_T_ and Persistence time of ASFV on fomites at environmental temperatures.

## Data Availability

The spreadsheet supporting the conclusions of this article is available in the https://doi.org/10.6084/m9.figshare.19706335.

## Abbreviations

ASF: African swine fever
ASFV: African swine fever virus
*D*_T_: Decimal reduction time at temperature T
DRT: Decimal reduction time
ANOVA: Analysis of variance
gof: Goodness-of-fit
RMSE: Root mean square error
PCR: Polymerase chain reaction assay
TCID: Tissue culture infective dose
HAD: Haemadsorption
